# Investigating the association of bed bugs with infectious diseases: A retrospective case-control study

**DOI:** 10.1016/j.heliyon.2021.e08107

**Published:** 2021-10-01

**Authors:** Johnathan M. Sheele, Claudia R. Libertin, Bobbi S. Pritt, Ewa M. Wysokinska, Jose E. Pietri

**Affiliations:** a Department of Emergency Medicine, Mayo Clinic, Jacksonville, FL, USA; b Division of Infectious Diseases, Mayo Clinic, Jacksonville, FL, USA; c Department of Laboratory Medicine and Pathology, Mayo Clinic, Rochester, MN, USA; d Division of Hematology and Medical Oncology, Mayo Clinic, Jacksonville, FL, USA; e Division of Basic Biomedical Sciences, Sanford School of Medicine, University of South Dakota, Vermillion, SD, USA

**Keywords:** Bacteremia, Cellulitis, *Cimex lectularius*, Coagulase-negative *Staphylococcus*, Eosinophil, Pneumonia

## Abstract

Bed bugs are common urban pests. Unlike many other blood-feeding human ectoparasites, bed bugs are not known to be vectors of human infectious diseases, but clinical and epidemiological studies to directly interrogate this link have been limited. Here, we aimed to determine whether bed bugs were associated with infectious diseases in a set of infested patients presenting to emergency departments (ED) in the greater Cleveland, OH area. We performed a retrospective case-control study involving 332 ED patients with bed bugs and 4,952 control patients, seen from February 1, 2011, through February 1, 2017. Cases and controls were matched by age, sex, and the presenting ED. Additionally, data were adjusted for ≥20 sociodemographic variables, triage data, and comorbidities in multivariable regression analyses. Seventeen laboratory values, ten different ED and inpatient diagnoses, chest radiographs, infectious disease consults, and blood cultures were examined. The odds of bed bug infestation were significantly higher for patients that had positive blood cultures, had blood cultures growing coagulase-negative *Staphylococcus*, were diagnosed with pneumonia, were diagnosed with cellulitis, received an infectious disease consult, received a chest radiograph, and had higher percentages of eosinophils in the blood (*P* < .05 for all). Additional investigations are needed to determine whether bed bugs directly contribute to disease by transmitting causative agents, whether bed bug exposure contributes secondarily contributes to infections, or whether these associations are better explained by other environmental and social determinants of health.

## 1 Introduction

The common bed bug, *Cimex lectularius* L., is an obligate blood-feeding ectoparasite that preferentially feeds on humans [1]. These insects have a cosmopolitan distribution, and infestations increasingly affect individuals of all socioeconomic backgrounds across urban environments [2, 3]. Bed bugs are associated with rashes (cimicosis), anemia, mental health disturbances, and rarely systemic allergic reactions [1, 4, 5, 6]. Associations between bed bugs and infectious diseases are less clear [7, 8, 9, 10, 11].

The ability of bed bugs to directly transmit human pathogenic microbes has been the subject of contentious debate, but the insects have not been shown to be vectors of infectious agents despite a lack of well-designed clinical investigations [7, 8, 9, 10, 11, 12]. Research to date has focused almost exclusively on the entomologic aspects of disease transmission. For example, multiple studies have tested the ability of bed bugs to transmit pathogenic agents in controlled laboratory settings, and bed bugs from the field have been screened for human pathogens [13, 14, 15, 16, 17, 18, 19]. A subset of these studies has yielded clinically significant results. Several human pathogenic microbes have been detected in bed bugs, including methicillin-resistant *Staphylococcus aureus* (MRSA), *Bartonella quintana*, *Burkholderia multivorans,* and hepatitis C virus [13, 14, 15, 16]. Further, the biological capacity to transmit microbes such as *B. quintana*, *Trypanosoma cruzi,* and *Borrelia recurrentis* has been shown in a laboratory setting [17, 18, 19]. However, the transmission of infectious agents from bed bugs to humans has never been demonstrated.

Given the prevalence of bed bugs, achieving a comprehensive understanding of the medical impacts of infestations is crucial to public health. A critical knowledge gap is that no published studies have examined the prevalence of infectious diseases among individuals with bed bug infestations. Such research is needed to interrogate possible links between bed bugs and infectious diseases more directly and has been encouraged by the US Centers for Disease Control and Prevention and US Environmental Protection Agency [20]. Indeed, case-control studies of human subjects have previously helped illuminate novel infectious risks associated with arthropods [e.g., the implication of insects in transmission of the agent of Buruli ulcer, [21, 22].

To further investigate whether bed bugs may be associated with any infectious diseases, we conducted a pilot case-control study to identify differences among urban emergency department (ED) patients with or without bed bugs in terms of laboratory findings and infectious disease diagnoses.

## 2 Materials and methods

After receiving institutional review board approval from University Hospitals Cleveland Medical Center (UHCMC), we conducted a retrospective chart review. Informed consent was waived for patients authorizing the use of their health records for research. Analyses examining allergic reactions, anemia, and mental health in the patient sets used in this study have been published previously [23, 24, 25].

### 2.1 Study population

Data were obtained from adult patients (age ≥18 years) who presented to 9 University Hospitals (UH) hospitals and freestanding emergency departments (EDs) located throughout the greater Cleveland-Akron, OH area from February 1, 2011, through February 1, 2017. UH EDs are located in a region of the United States with one of the highest rates of bed bug infestation and this is the site of several clinical studies of human subjects with bed bug infestation [3, 23, 24, 25]. There is no *International Classification of Diseases* code specific for bed bugs. Potential bed bug cases were identified by searching patient records for the keywords “bedbug,” “bed bug,” “Cimex,” or “lectularius.” A study investigator reviewed the clinical encounters and identified 332 patients with bed bugs based on previously described criteria [23, 24, 25]. Patients were included if they: 1) had a bed bug identified on them during the clinical encounter (most common), 2) self-reported having a bed bug infestation, or 3) had clinical suspicion of a bed bug infestation (e.g., based on EMS or ED staff reporting bed bugs without being able to capture one for identification). To maximize study power, patients with bed bugs were matched 1:15 to control patients with no documented history of bed bugs. Controls were matched based on age (±1 year at the time of the ED visit), sex, and the presenting ED location. Only the first ED visit in which a bed bug was identified was included in the data set for all cases. Patients who died in the ED were excluded from the analysis, though no deaths occurred in the bed bug infested patient group.

Only the first recorded set of ED vital signs and laboratory tests was obtained for each patient. We included only the results of ED cultures obtained in the ED, but we included consultations with infectious disease and dermatology specialists that occurred while patients were in the ED or were inpatients. Diagnoses established while patients were in the ED or inpatients were both included to capture a maximal number of diagnoses. These were provided in text format in the data set and were explored with keyword searches in Excel spreadsheet software (Microsoft Corp). Missing and erroneous laboratory data were not included in the final analysis.

### 2.2 Statistical analysis

The primary analyses consisted of multivariable logistic regression models to evaluate associations between patients with and without bed bugs (outcome) and selected primary predictors of interest (e.g., laboratory values, diagnoses, blood cultures), including the following adjustment variables: all available sociodemographic variables and triage data along with pertinent patient comorbidities which had <20% missing data. Each model included a single primary predictor (represented by the rows of Tables 1, 2, 3, and 4), along with the set of adjustment variables (provided as table footnote). All odds ratios (ORs) and 95% CIs were estimated. Reported P-values were two-sided and were adjusted for multiple comparisons (Bonferroni adjustment: adjusted P-value = raw p-value ∗ # of statistical tests presented in table) and P < 0.05 was used as the threshold for statistical significance. Multiple unadjusted (univariable) analyses were also conducted to explore associations in our dataset and are reported in Supplementary Tables 1–2. For these analyses, the continuous variables were summarized as mean and standard deviation. Categorical variables were summarized as frequency (percentage). Analyses were performed using JMP Pro 14 (SAS Institute Inc).

**Table 1:** Blood test results for patients with and without bed bugs.

Test	Multivariable Analysis^a^	
	bOR (95% CI)	*P* Value
White blood cell count, ×10^9^/L	.98 (.95–1.01)	>.99
Immature granulocyte count, ×10^9^/L	.12 (.007–2.19)	>.99
Neutrophil count, ×10^9^/L	.93 (.81–1.06)	>.99
Segmented neutrophil, ×10^9^/L	.96 (.86–1.07)	>.99
Eosinophil count, ×10^9^/L	2.22 (1.20–4.10)	.17
Eosinophils, %	1.12 (1.04–1.19)	.017
Basophil count, ×10^9^/L	2.38 (.03–180.03)	>.99
Monocyte count, ×10^9^/L	1.07 (.81–1.42)	>.99
Lymphocyte count, ×10^9^/L	1.06 (.99–1.14)	>.99
Erythrocyte sedimentation rate, mm/h	.97 (.92–1.03)	>.99
C-reactive protein, serum, mg/L	1.33 (.97–1.82)	>.99
Sodium, mEq/L	1.00 (.97–1.04)	>.99
Alanine aminotransferase, U/L	1.00 (.99–1.00)	>.99
Aspartate transaminase, U/L	1.00 (1.00–1.00)	>.99
Total bilirubin, mg/dL	.94 (.77–1.16)	>.99
Creatinine, mg/dL	1.03 (.95–1.12)	>.99
Blood cultures obtained	1.20 (.83–1.74)	>.99

Abbreviations: %, percent; mm/h, mEq/L, milliequivalents per liter; mg/dL, milligrams per deciliter; mg/L, milligrams per liter; mm/h, millimeters per hour; U/L, units per liter.

a Multivariable analysis adjusted for race (Black vs non-Black), emergency severity index (ESI), location before ED arrival (home vs nursing or rehabilitation facility vs physician office, clinic, surgery center, or inpatient elsewhere), ED disposition (admit/observation vs discharged, transferred, left against medical advice, or left without being seen), temperature, peripheral capillary oxygen saturation, respiratory rate, heart rate, mean arterial pressure, method of arrival (emergency medical service/police vs public transportation, walked, other vs private vehicle), body mass index (BMI), marital status (married/life partner vs single vs widowed, separated, divorced), health insurance (Medicaid, Medicare, private, or none/unknown), documented primary care physician (yes or no), ED triage screen for homicidal thoughts, suicidal ideation, or depression (yes to any vs no to all questions asked), tobacco use identified in triage or as an ED or inpatient diagnosis, hemoglobin (grams/deciliter), white blood cell count (×10^9^/L), serum glucose (mg/dL), serum creatinine (mg/dL), and serum bicarbonate (mEq/L).

b Odds ratios for bed bug infestation presented for a 1-unit increase in the test variable.

**Table 2:** Chest radiographs and infectious disease consults for patients with and without bed bugs.

Test	Multivariable Analysis^a^	
	bOR (95% CI)	*P* Value
Chest radiograph, No. (%)	1.52 (1.06–2.18)	.04
Infectious disease consult in the ED or inpatient, No. (%)	2.00 (1.14–3.53)	.04

a Multivariable analysis adjusted for race (Black vs non-Black), emergency severity index (ESI), location before ED arrival (home vs nursing or rehabilitation facility vs physician office, clinic, surgery center, or inpatient elsewhere), ED disposition (admit/observation vs discharged, transferred, left against medical advice, or left without being seen), temperature, peripheral capillary oxygen saturation, respiratory rate, heart rate, mean arterial pressure, method of arrival (emergency medical service/police vs public transportation, walked, other vs private vehicle), body mass index (BMI), marital status (married/life partner vs single vs widowed, separated, divorced), health insurance (Medicaid, Medicare, private, or none/unknown), documented primary care physician (yes or no), ED triage screen for homicidal thoughts, suicidal ideation, or depression (yes to any vs no to all questions asked), tobacco use identified in triage or as an ED or inpatient diagnosis, hemoglobin (grams/deciliter), white blood cell count (×10^9^/L), serum glucose (mg/dL), serum creatinine (mg/dL), serum bicarbonate (mEq/L), an ED or inpatient diagnosis of cognitive impairment (with the words: *dementia, Alzheimer's, cognitive impairment, intellectual disability,* or *Down's syndrome*), and a past or current diagnosis of human immunodeficiency virus (HIV) (yes or no/not documented).

b Odds ratios for bed bug infestation presented for those with vs without radiograph or consult.

**Table 3:** ED-only and ED + Inpatient diagnoses for patients with and without bed bugs.

Diagnosis	Multivariable Analysis^a^	
	bOR (95% CI)	*P* Value
**ED-Only Diagnoses**		
Pneumonia	1.91 (1.02–3.57)	.12
Cellulitis	3.02 (1.53–5.96)	.003
Sepsis	1.74 (.89–3.41)	.33
**ED + Inpatient Diagnoses**		
Pneumonia	1.89 (1.23–2.90)	.04
Bronchitis	1.40 (.71–2.75)	>.99
Cellulitis	3.47 (2.15–5.59)	.01
Abscess	1.37 (.73–2.57)	>.99
Sepsis	1.31 (.84–2.05)	>.99
Meningitis	1.67 (.18–15.22)	>.99
Hepatitis	1.01 (.41–2.51)	>.99
Endocarditis	1.61 (.16–15.85)	>.99
Bacteremia	2.46 (1.19–5.08)	.2
Bacteremia risk factors^c^	.93 (.61–1.40)	>.99

Abbreviations: CI, confidence intervals; ED, emergency department; OR, odds ratio.

a Multivariable analysis adjusted for race (Black vs non-Black), emergency severity index (ESI), location before ED arrival (home vs nursing or rehabilitation facility vs physician office, clinic, surgery center, or inpatient elsewhere), ED disposition (admit/observation vs discharged, transferred, left against medical advice, or left without being seen), temperature, peripheral capillary oxygen saturation, respiratory rate, heart rate, mean arterial pressure, method of arrival (emergency medical service/police vs public transportation, walked, other vs private vehicle), body mass index (BMI), marital status (married/life partner vs single vs widowed, separated, divorced), health insurance (Medicaid, Medicare, private, or none/unknown), documented primary care physician (yes or no), ED triage screen for homicidal thoughts, suicidal ideation, or depression (yes to any vs no to all questions asked), an ED or inpatient diagnosis of illicit drug use (with the words: *polysubstance, substance abuse, cocaine, heroin, marijuana, methamphetamine, PCP, phencyclidine, cannabis,* or *inhalant-use disorder*), tobacco use identified in triage or as an ED or inpatient diagnosis, a past or current diagnosis of dialysis, an ED or inpatient diagnosis of cognitive impairment (with the words: *dementia, Alzheimer's, cognitive impairment, intellectual disability,* or *Down's syndrome*), and a past or current diagnosis of human immunodeficiency virus (HIV) (yes or no/not documented).

b Odds ratios for bed bug infestation presented for those with vs without the diagnosis.

c Defined as having an ED or inpatient diagnosis that included the terms *catheter, device, prosthetic, shunt, implant, intravascular, graft, PICC, central venous, line, osteomyelitis, endocarditis, septic arthritis,* or *septic joint*.

**Table 4:** Blood cultures obtained in the ED.

Test	Multivariable Analysis^a^	
	bOR (95% CI)	*P* Value
Growing any bacteria	2.77 (1.30–5.89)	.024
Growing coagulase-negative *Staphylococcus*	4.22 (1.74–10.23)	.006
Growing *Staphylococcus aureus*	2.32 (.30–17.95)	>.99

Abbreviations: CFU, colony forming unit; ED, emergency department; OR, odds ratio.

a Multivariable analysis adjusted for race (Black vs non-Black), emergency severity index (ESI), location before ED arrival (home vs nursing or rehabilitation facility vs physician office, clinic, surgery center, or inpatient elsewhere), ED disposition (admit/observation vs discharged, transferred, left against medical advice, or left without being seen), temperature, peripheral capillary oxygen saturation, respiratory rate, heart rate, mean arterial pressure, method of arrival (emergency medical service/police vs public transportation, walked, other vs private vehicle), body mass index (BMI), marital status (married/life partner vs single vs widowed, separated, divorced), health insurance (Medicaid, Medicare, private, or none/unknown), documented primary care physician (yes or no), ED triage screen for homicidal thoughts, suicidal ideation, or depression (yes to any vs no to all questions asked), tobacco use identified in triage or as an ED or inpatient diagnosis, a past or current diagnosis of dialysis, an ED or inpatient diagnosis of cognitive impairment (with diagnosis of: *dementia, Alzheimer's, cognitive impairment, intellectual disability,* or *Down's syndrome*), and a past or current diagnosis of human immunodeficiency virus (HIV) (yes or no/not documented), site where the blood culture was obtained (peripheral site versus a central venous line (CVL), peripheral inserted central catheter (PICC), or mediport), and having an ED or inpatient diagnosis with the words: catheter, device, prosthesis or prosthetic, shunt, implant, intravascular, graft, PICC, CVL, line, osteomyelitis, endocarditis, septic arthritis, or septic joint.

b Odds ratios for bed bug infestation presented for those with vs without a positive culture.

## 3 Results

### 3.1 Patient characteristics

Patient characteristics and triage data for the 332 patients with bed bugs and 4,952 controls are summarized in Supplementary Tables 1–2. In the cohort, 3,609 (68.3%) of patients were aged 55 years or older, 3,017 (57.1%) were women, and 2,980 (56.4%) were Black.

### 3.2 Blood tests

The odds of bed bug infestation were significantly higher for patients with higher eosinophil percentages (OR, 1.12 [95% CI, 1.04–1.19]; *P* = .017) (Table 1). Additionally, the odds of bed bug infestation were higher for patients with higher eosinophil counts, though this did not reach statistical significance in the multivariable regression model (OR, 2.22 [95% CI, 1.20–4.10]; *P* = .17). The same associations were also identified in our unadjusted analyses (P < .05, Supplementary Table 2).

### 3.3 Radiography

The odds of bed bug infestation were significantly higher in patients that received a chest radiograph (OR, 1.52 [95% CI, 1.06–2.18]; *P* = .04) (Table 2). The same association was also found in our unadjusted analyses (P < .05, Supplementary Table 2).

### 3.4 Consults

The odds of bed bug infestation were significantly higher for patients that received an infectious disease consult (OR, 2.00 [95% CI, 1.14–3.53]; *P* = .04) (Table 2). The same association was also seen in our unadjusted analysis (P < .05, Supplementary Table 2).

### 3.5 ED diagnoses

The odds of bed bug infestation were significantly higher for patients diagnosed with cellulitis in the ED (OR, 3.02 [95% CI, 1.53–5.96]; *P* = .003) (Table 3). The odds of bed bug infestation were also higher for patients diagnosed with pneumonia in the ED, though this did not reach statistical significance in the multivariable regression model (OR, 1.91 [95% CI, 1.02–3.57]; *P* = .12) (Table 3). Our unadjusted analyses also found associations between bed bug infestation and ED diagnoses of cellulitis and pneumonia (P < .05, Supplementary Table 2).

### 3.6 ED plus inpatient diagnoses

The odds of bed bug infestation were also significantly higher for patients with an ED or inpatient diagnosis of cellulitis (OR, 3.47 [95% CI, 2.57–5.59]; *P* = .01) (Table 3). Similarly, the odds of bed bug infestation were significantly higher for patients with an ED or inpatient diagnosis of pneumonia (OR, 1.89 [95% CI, 1.23–2.90]; *P* = .04) (Table 3). Our unadjusted analyses also found associations between bed bug infestation and ED or inpatient diagnoses of cellulitis and pneumonia (P < .05, Supplementary Table 2).

No bed bug infested or uninfested patients had a diagnosis that included the terms “*Ehrlichia*,” “*Anaplasma*,” “arbovirus,” “arboviral,” “babesiosis,” “*Yersinia*,” “Lyme,” “*Rickettsia*,” “*Bartonella*,” “*Burkholderia*,” “*Borrelia*,” “cat scratch,” “tularemia,” “*Francisella*,” “lice,” “typhus,” “Q fever,” or “Trypanosoma” reflecting the extreme rarity of these diagnoses in northeast Ohio. Additionally, no patient with bed bugs underwent a lumbar puncture for a neurologic condition, and no patients with bed bugs were diagnosed with vasculitis or impetigo compared to 21 and two patients without bed bugs that had vasculitis and impetigo, respectively. There was no bed bug infested patient diagnosed with encephalitis compared to three patients without bed bugs. Two bed bug patients were diagnosed with endocarditis, with one growing CoNS and the other not having blood cultures drawn in the ED. This compared to six uninfested patients being diagnosed with endocarditis, of which two grew *Staphylococcus aureus*, one grew *Streptococcus pneumoniae*, one had no growth in the blood culture, and two had no blood cultures drawn in the ED.

### 3.7 Blood cultures in the ED

The odds of bed bug infestation were not different for patients that had or did not have blood cultures drawn in the ED (OR, 1.20 [95% CI, .83–1.74]; *P* > .99) (Table 1). However, the odds of bed bug infestation were significantly higher for patients that had a positive bacterial blood culture (OR, 2.77 [95% CI, 1.30–5.89]; *P* = .024) and that had a blood culture growing CoNS (OR, 4.22 [95% CI, 1.74–10.23]; *P* = .006). These associations was also apparent in our unadjusted analyses (P < .05, Supplementary Table 2).

Three of 17 patients with bed bugs and blood cultures growing CoNS (17.6%) had two or more of their cultures growing CoNS, and the rest showed CoNS in a single culture. Two of 44 patients without bed bugs and blood cultures growing CoNS (4.5%) had two or more of their cultures growing CoNS, and the rest showed CoNS in a single culture (Supplementary Table 2). There were four patients with bed bugs and eight patients without bed bugs who had an ED or inpatient diagnosis of bacteremia plus an ED blood culture growing CoNS. All patients with an ED or inpatient diagnosis of bacteremia and a positive CoNS culture had a single ED blood culture positive for CoNS, except for one patient with bed bugs who had both of their two cultures positive for CoNS. All patients diagnosed with bacteremia who had at least 1 CoNS-positive culture were admitted to the hospital. There were no differences in antibiotic sensitivities of bacterial cultures obtained from the blood of patients with or without bed bug infestation (Supplementary Table 3).

## 4 Discussion

Long-standing dogma is that bed bugs do not transmit infectious organisms to humans; however, no rigorous clinical investigation supports this assertion. Our study was not designed to support or refute pathogen transmission by bed bugs; instead, our reported associations suggest that further investigation should be conducted unencumbered by anchoring bias. Similar case-control studies of bed bug infested patients are lacking, and the absence of a specific ICD code for bed bugs hamper clinical research in the field.

Our study has a number of limitations that are further discussed below, but a significant strength is that our multivariable analyses accounted for at least 20 social variables, demographic information, triage data, and comorbidities, plus the dataset matched cases and controls on three additional variables. Therefore, the existence of major unaccounted socioeconomic or demographic variables to explain the significant associations we uncovered is less likely.

Most of the variables we explored showed no significant differences between bed bug infested and uninfested patients, with a few notable exceptions. Patients with higher eosinophil percentages in the blood were more likely to be infested with bed bugs. Eosinophilia has been reported previously in a small number of case reports, and it has been observed in skin biopsies from patients with bed bugs [26, 27, 28, 29]. Similarly, mice that were fed on by bed bugs had higher eosinophil, basophil, and monocyte counts [30], and eosinophilia have been reported with *Pediculus humanus capitis* (head lice) and *Pediculus humanus corporis* (body lice) infestations, consistent with our findings [31].

Patients diagnosed with pneumonia were more likely to be infested with bed bugs. Intriguingly, recent research revealed that bed bug infestations lead to elevated levels of histamine in human homes due to the release of this molecule in the feces of the insects [32]. Histamine has a dose-dependent effect on human health and can cause respiratory symptoms, including bronchospasm. It is unclear whether bed bug associated histamine exposure could result in respiratory symptoms or non-specific chest radiographic findings leading to diagnoses of pneumonia.

Patients diagnosed with cellulitis were also more likely to be infested with bed bugs, although our study could not exclude the possibility that clinicians were misdiagnosing cimicosis as cellulitis when no true bacterial skin and soft tissue infection existed. Bed bugs have been previously cited as causing cellulitis, although no experimental evidence has been reported until now [1, 4]. Nevertheless, in support of our results, other insect bites and hematophagous insect feedings have been associated with skin and soft tissue infections [33, 34].

Patients with positive blood cultures, particularly blood cultures growing CoNS, were more likely to be infested with bed bugs, although it is not clear whether they had higher rates of pathological bacteremia or whether these were false-positive results from environmental contamination. Further research is needed to explore this association. However, bed bugs could theoretically cause either cellulitis or bacteremia by direct inoculation while feeding, by mechanical contamination of the feeding site, or indirectly if the host self-inoculated skin bacteria into a pruritic insect feeding site.

At least four gram-positive bacteria species are known to be associated with bed bugs (*Staphylococcus arlettae*, *Staphylococcus epidermidis*, *Kocuria kristinae*, and *Micrococcus* spp) [35], but of these, only *Staphylococcus* bacteria are a frequent cause of skin and soft tissue infections in humans. *Staphylococcus* is extremely common in the environment, and different species, including MRSA, have been detected in association with multiple field-collected bed bug samples [16]. MRSA can survive internally in the bed bug gut for up to nine days, but shedding the bacteria in the feces or from mouthparts has not been thoroughly tested [36]. Cultures of the proboscis from laboratory-raised *C lectularius* have not grown *Staphylococcus* [37], and a vector-borne transmission cycle is not known for any *Staphylococcus* species. However, a 1936 report shows that *C. lectularius* can transmit hemolytic *Staphylococcus* to rabbits, mice, and guinea pigs and that the bacteria are detectable in insect salivary glands for up to 15 days [38].

Some bed bug populations have recently been found to harbor an uncharacterized species of *Rickettsia* [39]. Human pathogenic *Rickettsia* can cause fever, leukopenia, transaminitis, and hyponatremia. Our study did not find evidence that these were associated with bed bugs in our patient cohort. Bed bugs have also been studied for their association with hepatitis viruses [12, 13] but bed bugs were not associated with a diagnosis of hepatitis or with transaminitis in our patient cohort.

Although no large-scale viromic investigations of bed bugs have been published [12], the insects are not known to be vectors of arboviruses. Some human arboviruses can cause symptoms such as encephalitis, fever, hepatitis, and renal insufficiency but none of these were significantly associated with bed bugs in our patient cohort.

### 4.1 Limitations

It is essential to recognize that we do not know how long our infested patients had their bed bugs which is critical for exploring associations with possible vector-borne diseases, some of which can be mild (or asymptomatic) and transient, and may not have prompted an ED evaluation. By only studying an ED population, our study is biased towards a more acutely ill patient whose presentation to the ED may not have been directly or indirectly related to bed bugs.

Patients could have been misclassified as having bed bugs, or they may have picked up an insect within the ED (and thus not actually been infested with bed bugs), but those scenarios are unlikely given previous experience with bed bug detection and identification at UHCMC [40, 41, 42, 43, 44]. Our cohort is likely biased toward patients with large, long-term bed bug infestations that are easier to detect, which would also limit our ability to identify acute infections associated with bed bugs. If bed bugs were able to transmit infectious agents that cause acute disease in humans, infection would likely occur early during a new infestation or when a person first enters an infested environment and thus unlikely to be identified in our dataset.

The study took place in the greater Cleveland area of Ohio, which has a high burden of bed bugs [3]. A bed bug is found approximately every two days in UHCMC, an academic tertiary care center in downtown Cleveland, and about every five days within the UHCMC ED [42, 43]. Collecting data from a primarily urban area in northeast Ohio could miss vector-borne diseases transmitted seasonally or otherwise geographically restricted outside of our studied location, as most known vector-borne diseases are uncommon in northeast Ohio and our study was not powered to detect the occurrence of rare infections.

Many of the infested and uninfested patients lived in Cleveland and East Cleveland, two of the most economically disadvantaged cities in Ohio. Indeed, a previous study of UHCMC ED patients found that bed bug infestation was not associated with socioeconomic status, as most patients, whether infested with bed bugs or not, had household incomes <$25,000/year [43]. Therefore, while our approach considered many demographic and socioeconomic variables, our findings may not be generalizable to a more socioeconomically, racially, and geographically diverse population. A lack of comprehensive inpatient and outpatient testing and longitudinal patient follow-up likely missed some infections and pertinent testing and diagnoses. Lastly, we only examined data from cultures performed from the ED, so diagnostic evaluations performed as an outpatient, any inpatient studies which resulted after the patient was discharged, or the results from subsequent hospitalizations would not have been included in our dataset. Thus, negative associations from our studies should not be used to justify the exclusion of bed bugs as vectors of disease without proper context.

## 5 Conclusions

Bed bug infestation was significantly more likely in patients diagnosed with cellulitis and with higher percent blood eosinophils. Infestation was also more likely in patients diagnosed with pneumonia, with positive blood cultures, and with blood cultures growing CoNS. Additional studies in other patient populations and geographical regions are needed to determine the cause and significance of these associations.

## Declarations

### Author contribution statement

Johnathan M. Sheele: Conceived and designed the experiments; Performed the experiments; Analyzed and interpreted the data; Contributed reagents, materials, analysis tools or data; Wrote the paper.

Claudia R. Libertin, Bobbi S. Pritt, Ewa M. Wysokinska, Jose E. Pietri: Conceived and designed the experiments; Wrote the paper.

### Funding statement

This research did not receive any specific grant from funding agencies in the public, commercial, or not-for-profit sectors.

### Data availability statement

The data that has been used is confidential.

### Competing interest statement

The authors declare no conflict of interest.

### Additional information

No additional information is available for this paper.

## References

[1] Goddard J., deShazo R. (2009). Bed bugs (*Cimex lectularius*) and clinical consequences of their bites. J. Am. Med. Assoc..

[2] Wu Y., Tracy D.M., Barbarin A.M., Barbu C.M., Levy M.Z. (2014). A door-to-door survey of bed bug (Cimex lectularius) infestations in row homes in Philadelphia, Pennsylvania. Am. J. Trop. Med. Hyg..

[3] Jones S.C. (2021). Multitude and spread of bed bugs (Cimex lectularius) throughout Ohio (USA) revealed by surveys of pest management industry. Insects.

[4] Doggett S.L., Dwyer D.E., Penas P.F., Russell R.C. (2012). Bed bugs: clinical relevance and control options. Clin. Microbiol. Rev..

[5] Munoz-Price L.S., Safdar N., Beier J.C., Doggett S.L. (2012). Bed bugs in healthcare settings. Infect. Control Hosp. Epidemiol..

[6] Susser S.R., Perron S., Fournier M., Jacques L., Dennis G., Tessier F., Roberge P. (2012). Mental health effects from urban bed bug infestation (Cimex lectularius L): a cross-sectional study. BMJ Open.

[7] Ho D., Lai O., Glick S., Jagdeo J. (2016). Lack of evidence that bedbugs transmit pathogens to humans. J. Am. Acad. Dermatol..

[8] Lai O., Ho D., Glick S., Jagdeo J. (2016). Bed bugs and possible transmission of human pathogens: a systematic review. Arch. Dermatol. Res..

[9] Zorrilla-Vaca A., Silva-Medina M.M., Escandón-Vargas K. (2015). Bedbugs, Cimex spp.: their current world resurgence and healthcare impact. Asian Pac. J. Trop Dis..

[10] Delaunay P., Blanc V., Del Giudice P. (2011). Bedbugs and infectious diseases. Clin. Infect. Dis..

[11] Pietri J.E. (2020). Case not closed: arguments for new studies of the interactions between bed bugs and human pathogens. Am. J. Trop. Med. Hyg..

[12] Adelman Z., Miller D.M., Myles M.K. (2013). Bed bugs and infectious diseases: a case for the arboviruses. PLoS Pathog..

[13] Ling J., Vinnersten T.P., Hesson J.C., Bohlin J., Roligheten E., Holmes E.C., Pettersson J.H.O. (2020). Identification of hepatitis C virus in the common bed bugs- a potential, but uncommon route for HCV infection?. Emerg. Microb. Infect..

[14] Angelakis E., Socolovschi C., Raoult D. (2013). Bartonella quintana in Cimex hemipterus, Rwanda. Am. J. Trop. Med. Hyg..

[15] Saenz V.L., Maggi R.G., Breitschwerdt E.B., Kim J., Vargo E.L., Schal C. (2013). Survey of Bartonella spp. in U.S. bed bugs detects Burkholderia multivorans but not Bartonella. PLoS One.

[16] Lowe C.F., Romney M.G. (2011). Bedbugs as vectors for drug-resistant bacteria. Emerg. Infect. Dis..

[17] Salazar R., Castillo-Neyra R., Tustin A.W., Borrini-Mayori K., Naquira C., Levy M.Z. (2015). Bed bugs (Cimex lectularius) as vectors of Trypanosoma cruzi. Am. J. Trop. Med. Hyg..

[18] Leulmi H., Bitam I., Berenger J.M. (2015). Competence of Cimex lectularius bed bugs for the transmission of Bartonella quintana, the agent of trench fever. PLoS Neglected Trop. Dis..

[19] El Hamzaoui B., Laroche M., Bechah Y., Berenger J.M., Parola P. (2019). Testing the competence of Cimex lectularius bed bugs for the transmission of Borrelia recurrentis, the agent of relapsing fever. Am. J. Trop. Med. Hyg..

[20] US Centers for Disease Control and Prevention and US Environmental Protection Agency (2010).

[21] Quek T.Y.J., Athan E., Henry M.J., Pasco J.A., Redden-Hoare J., Hughes A., Johnson P.D.R. (2007). Risk factors for Mycobacterium ulcerans infection, Southeastern Australia. Emerg. Infect. Dis..

[22] Pouillot E., Matias G., Wondje C.M., Portaels F., Valin N., Ngos F., Njikap A., Marsoiller L., Fontanet A., Eyangoh S. (2007). Risk factors for buruli ulcer: a case control study in Cameroon. PLoS Neglected Trop. Dis..

[23] Sheele J.M., Pritt B.S., Libertin C.R., Wysokinska E.M. (2020). Bed bugs are associated with anemia. Am. J. Emerg. Med..

[24] Sheele J.M. (2021). Association between bed bugs and allergic reactions. Parasite Immunol..

[25] Sheele J.M. (2021). Associations between bed bugs and mental illness among emergency department patients. Cureus.

[26] Scarupa M.D., Economides A. (2006). Bedbug bites masquerading as urticaria. J. Allergy Clin. Immunol..

[27] Phan C., Brunet-Possenti F., Marinho E., Petit A. (2016). Systemic reactions caused by bed bug bites. Clin. Infect. Dis..

[28] Kennedy J.L., Jeffus S.K., Platts-Mills T.A. (2013). Seventeen-year-old girl hospitalized for localized swelling, pruritus, tenderness, and lymphatic streaking with eosinophilia. J. Allergy Clin. Immunol. Pract..

[29] deShazo R.D., Feldlaufer M.F., Mihm M.C., Goddard J. (2012). Bullous reactions to bedbug bites reflect cutaneous vasculitis. Am. J. Med..

[30] Abdel-Hamid Y.M., Soliman M.I. (2010). Hematological changes in mice exposed to biting of the bedbug: Cimex lectularius L. (Hemiptera: cimicidae). J. Egypt. Soc. Parasitol..

[31] Woodruff C.M., Chang A.Y. (2019). More than skin deep: severe iron deficiency anemia and eosinophilia associated with pediculosis capitis and corporis infestation. JAAD Case Rep..

[32] DeVries Z.C., Santangelo R.G., Barbarin A.M., Schal C. (2018). Histamine as an emergent indoor contaminant: accumulation and persistence in bed bug infested homes. PLoS One.

[33] Derlet R.W., Richards J.R. (2003). Cellulitis from insect bites: a case series. Cal. J. Emerg. Med..

[34] Kontoyiannis P.D., Koons G.L., Hicklen R.S., Mikos A.G., Kontoyiannis D.P. (2019). Insect bite-associated invasive fungal infections. Open Forum Infect. Dis..

[35] Cockburn C., Amoroso M., Carpenter M. (2013). Gram-positive bacteria isolated from the common bed bug, Cimex lectularius L. Entomol. Am..

[36] Barbarin A.M., Hu B., Nachamkin I., Levy M.Z. (2014). Colonization of Cimex lectularius with methicillin-resistant Staphylococcus aureus. Environ. Microbiol..

[37] Reinhardt K., Naylor R.A., Siva-Jothy M.T. (2005). Potential sexual transmission of environmental microbes in a traumatically inseminating insect. Ecol. Entomol..

[38] Epstein G., Exemplarskaya E., Silvers I., Babekova O. (1936). Bedbugs as transmitters of hemolytic staphylococci to experimental animals. G. Batteriol. Immunol..

[39] Potts R., Molina A., Sheele J.M., Pietri J.E. (2020). Molecular detection of Rickettsia infection in field-collected bed bugs. New Microb. New Infect..

[40] Sheele J.M., Barrett E., Dash D., Ridge G.E. (2017). Analysis of the life stages of Cimex lectularius captured within a medical centre suggests that the true numbers of bed bug introductions are under-reported. J. Hosp. Infect..

[41] Sheele J.M., Barrett E., Farhan O., Morris N. (2017). Analysis of bed bug (Cimex lectularius) introductions into an academic medical center. Infect. Control Hosp. Epidemiol..

[42] Sheele J.M., Crandall C.J., Chang B.F., Arko B.L., Dunn C.T., Negrete A. (2019). Characteristics of bed bug infested patients in the emergency department. Emerg. Med. Int..

[43] Sheele J.M., Crandall C.J., Chang B.F., Arko B.L., Dunn C.T., Negrete A. (2019). Risk factors for bed bugs among urban emergency department patients. J. Community Health.

[44] Totten V., Charbonneau H., Hoch W., Shah S., Sheele J.M. (2016). The cost of decontaminating an ED after finding a bed bug: results from a single academic medical center. Am. J. Emerg. Med..

