# Supplementary material for: Investigating the association of bed bugs with infectious diseases: A retrospective case-control study
Source: Heliyon. 2021 Oct 1;7(10):e08107. doi: 10.1016/j.heliyon.2021.e08107 (PMC8569396; doi:10.1016/j.heliyon.2021.e08107)
Supplement: supplement 1 [file mmc1.docx]

**Supplementary Table 1. Patient Characteristics (N=5,284)**

| **Characteristic** | **Bed Bugs (n=332)** | **No Bed Bugs (n=4,952)** | ***P* Value** |
| --- | --- | --- | --- |
| Sex, No. (%) |  |  |  |
| Male | 143 (43.1) | 2124 (42.9) | NA |
| Female | 189 (56.9) | 2828 (57.1) |  |
| Age, No. (%) |  |  |  |
| ≤40 | 47 (14.2) | 703 (14.2) |  |
| 41-68 | 171 (51.5) | 2,548 (51.5) | NA |
| ≥69 | 114 (34.3) | 1,701 (34.3) |  |
| Race, No. (%) |  |  | <.001 |
| Other than Black | 57 (17.2) | 2,233 (45.4) |  |
| Black/African American | 274 (82.8) | 2,684 (54.6) |  |
| Marital status, No. (%) |  |  | <.001 |
| Married or life partner | 47 (14.3) | 1,659 (33.8) |  |
| Single | 190 (57.8) | 1,990 (40.5) |  |
| Separated | 4 (1.2) | 74 (1.5) |  |
| Widowed | 41 (12.5) | 688 (14.0) |  |
| Divorced | 47 (14.3) | 498 (10.1) |  |
| Health insurance, No. (%) |  |  | .02 |
| Medicaid | 26 (7.8) | 229 (4.6) |  |
| Medicare | 98 (29.5) | 1,322 (26.7) |  |
| Private | 177 (58.3) | 2,949 (59.6) |  |
| Unknown or none | 31 (9.3) | 452 (9.1) |  |
| Location before ED visit, No. (%) |  |  | <.001 |
| Home | 323 (97.3) | 4,442 (89.7) |  |
| Nursing or rehabilitation facility | 5 (1.5) | 140 (2.8) |  |
| Physician office, clinic, surgery center, inpatient at another acute care facility | 2 (0.6) | 244 (4.9) |  |
| Unknown | 2 (0.6) | 126 (2.5) |  |
| Method of ED arrival, No. (%) |  |  | <.001 |
| Emergency medical service, helicopter, police | 166 (50.0) | 1,655 (33.4) |  |
| Private vehicle | 98 (29.5) | 3,036 (61.3) |  |
| Public transport | 1 (0.3) | 44 (0.9) |  |
| Walked | 3 (0.9) | 124 (2.5) |  |
| Other or missing data | 64 (19.3) | 93 (1.9) |  |

Abbreviation: %, percent; ED, emergency department; No., number; SD, standard deviation
