# Supplementary material for: Investigating the association of bed bugs with infectious diseases: A retrospective case-control study
Source: Heliyon. 2021 Oct 1;7(10):e08107. doi: 10.1016/j.heliyon.2021.e08107 (PMC8569396; doi:10.1016/j.heliyon.2021.e08107)
Supplement: Supplement 2 [file mmc2.docx]

**Supplementary Table 2. Unadjusted (univariable) analysis of patients with and without bed bugs**

|  | **Bed Bugs,**  **Mean (SD)**  **or No. (%)** | **No Bed Bugs,**  **Mean (SD)**  **or No. (%)** | **Unadjusted Analysis** | |  |
| --- | --- | --- | --- | --- | --- |
| **Variable** |  |  | **OR (95% CI)** | ***P* Value** |  |
| **Laboratory values** |  |  |  |  |  |
| White blood cell count, ×10^9^/L | 9.27 (5.20)  (n=274) | 9.16 (4.91)  (n=2,825) | 1.00 (0.98-1.03) | .71 |  |
| Absolute neutrophil count, ×10^9^/L | 11.22 (7.28)  (n=21) | 11.21 (11.19)  (n=145) | 1.00 (0.96-1.04) | >.99 |  |
| Immature granulocyte count, ×10^9^/L | 0.06 (0.07)  (n=116) | 0.08 (0.36)  (n=1,541) | 0.55 (0.12-2.59) | .45 |  |
| Immature granulocytes, % | 0.61 (0.67)  (n=119) | 0.68 (1.47)  (n=1,571) | 0.96 (0.81-1.13) | .60 |  |
| Neutrophil count, ×10^9^/L | 6.20 (3.70)  (n=241) | 6.16 (3.67)  (n=2,452) | 1.00 (0.97-1.04) | .87 |  |
| Neutrophils, % | 66.28 (15.15)  (n=241) | 66.43 (13.99)  (n=2,453) | 1.00 (0.99-1.01) | .88 |  |
| Segmented neutrophil, ×10^9^/L | 9.75 (6.93) (n=21) | 9.19 (7.15) (n=145) | 1.01 (0.95-1.08) | .74 |  |
| Segmented neutrophil count, % | 62.62 (22.30)  (n=21) | 61.32 (20.13)  (n=145) | 1.00 (0.98-1.03) | .78 |  |
| Eosinophil count, ×10^9^/L | 0.19 (0.31)  (n=236) | 0.15 (0.19)  (n=2,430) | 1.96 (1.21-3.17) | .006 |  |
| Eosinophils, % | 2.69 (4.36)  (n=217) | 1.98 (2.07)  (n=2,292) | 1.09 (1.04-1.14) | <.001 |  |
| Basophil count, ×10^9^/L | 0.04 (0.03)  (n=245) | 0.04 (0.03)  (n=2,539) | 0.26 (0.003-23.79) | .56 |  |
| Basophils, % | 0.44 (0.33)  (n=234) | 0.47 (0.42)  (n=2,431) | 0.85 (0.57-1.28) | .44 |  |
| Monocyte count, ×10^9^/L | 0.76 (0.64)  (n=255) | 0.72 (0.44)  (n=2,582) | 1.19 (0.94-1.50) | .14 |  |
| Monocytes, % | 8.61 (3.76)  (n=255) | 8.25 (3.39)  (n=2,582) | 1.03 (0.99-1.06) | .11 |  |
| Lymphocyte count, ×10^9^/L | 1.89 (2.99)  (n=255) | 1.82 (1.27)  (n=2,582) | 3.11 (0.11-85.90) | .50 |  |
| Lymphocytes, % | 21.94 (13.81)  (n=255) | 22.41 (12.95)  (n=2,582) | 1.00 (0.99-1.01) | .58 |  |
| Erythrocyte sedimentation rate, mm/h | 44.96 (36.19)  (n=23) | 38.83 (34.30)  (n=155) | 1.00 (0.99-1.02) | .43 |  |
| C-reactive protein, serum, mg/L | 8.97 (11.70)  (n=26) | 4.30 (6.21)  (n=155) | 1.07 (1.02-1.12) | .006 |  |
| Sodium, mEq/L | 138.45 (5.96)  (n=271) | 138.78 (4.10)  (n=2,862) | 0.98 (0.95-1.01) | .21 |  |
| Alanine aminotransferase, U/L | 31.26 (22.28)  (n=184) | 38.02 (90.18)  (n=1,700) | 1.00 (0.99-1.00) | .27 |  |
| Aspartate transaminase, U/L | 41.10 (42.00)  (n=184) | 42.50 (121.05)  (n=1,700) | 1.00 (1.00-1.00) | .87 |  |
| Albumin, g/dL | 3.41 (0.66)  (n=202) | 3.59 (0.59)  (n=1,963) | 0.62 (0.50-0.78) | <.001 |  |
| Anion gap, mEq/L | 14.55 (4.40)  (n=269) | 13.37 (3.31)  (n=2,861) | 1.08 (1.05-1.12) | <.001 |  |
| Total bilirubin, mg/dL | 0.75 (0.83) (n=184) | 0.73 (1.16)  (n=1,703) | 1.01 (0.89-1.15) | .84 |  |
| Direct bilirubin, mg/dL | 0.29 (0.36)  (n=61) | 0.37 (1.04)  (n=352) | 0.88 (0.57-1.36) | .55 |  |
| Creatinine, mg/dL | 1.87 (2.30)  (n=271) | 1.34 (1.44)  (n=2,860) | 1.15 (1.09-1.22) | <.001 |  |
| Glomerular filtration rate, mL/min | 46.98 (18.20)  (n=272) | 52.73 (14.10)  (n=2,862) | 0.98 (0.97-0.99) | <.001 |  |
| Albumin:globulin ratio | 0.90 (0.28)  (n=182) | 1.05 (0.32)  (n=1,697) | 0.17 (0.10-0.30) | <.001 |  |
| Chest radiograph, No. (%) | 198 (59.6) | 1,674 (33.8) | 2.89 (2.31-3.63) | <.001 |  |
| Infectious disease consult in the ED or inpatient, No. (%) | 23 (6.9) | 101 (2.0) | 3.58 (2.24-5.70) | <.001 |  |
| **ED diagnoses-only** |  |  |  |  |  |
| Pneumonia | 17 (5.1) | 93 (1.9) | 2.82 (1.66-4.79) | <.001 |  |
| Cellulitis | 18 (5.4) | 76 (1.5) | 3.68 (2.17-6.23) | <.001 |  |
| Abscess | 6 (1.8) | 79 (1.6) | 1.14 (0.49-2.62) | .77 |  |
| Sepsis | 17 (5.1) | 79 (1.6) | 3.33 (1.95-5.69) | <.001 |  |
| **ED + Inpatient diagnoses-only** |  |  |  |  |  |
| Pneumonia | 44 (13.3) | 273 (5.5) | 2.62 (1.86-3.68) | <.001 |  |
| Abscess | 19 (5.7) | 168 (3.4) | 1.73 (1.06-2.82) | .03 |  |
| Cellulitis | 39 (11.8) | 161 (3.3) | 3.96 (2.74-5.73) | <.001 |  |
| Viral illness | 14 (4.2) | 102 (2.1) | 2.09 (1.18-3.70) | .01 |  |
| Diarrhea | 22 (6.6) | 181 (3.7) | 1.87 (1.18-2.95) | .007 |  |
| Arthritis or arthralgia | 26 (7.8) | 320 (6.5) | 1.22 (0.81-1.86) | .33 |  |
| Meningitis | 1 (0.3) | 12 (0.2) | 1.24 (0.16-9.59) | .83 |  |
| Encephalitis | 0 (0) | 3 (0.1) | NA | NA |  |
| Seizure | 24 (7.2) | 179 (3.6) | 2.08 (1.33-3.23) | .001 |  |
| Altered mental status | 48 (14.5) | 307 (6.2) | 2.56 (1.84-3.54) | <.001 |  |
| Hepatitis | 7 (2.1) | 53 (1.1) | 1.99 (0.90-4.41) | .09 |  |
| HIV | 6 (1.8) | 46 (0.9) | 1.96 (0.83-4.63) | .12 |  |
| Sepsis | 37 (11.1) | 253 (5.1) | 2.33 (1.62-3.35) | <.001 |  |
| Bacteremia | 15 (4.5) | 61 (1.2) | 3.79 (2.13-6.75) | <.001 |  |
| Endocarditis | 2 (0.6) | 6 (0.1) | 5.00 (1.00-24.85) | .049 |  |
| *Clostridioides difficile* | 7 (2.1) | 49 (1.0) | 2.16 (0.97-4.80) | .06 |  |
| Osteomyelitis | 3 (0.9) | 28 (0.6) | 1.60 (0.48-5.30) | .44 |  |
| Urinary tract infection | 18 (5.4) | 201 (4.1) | 1.35 (0.83-2.22) | .23 |  |
| Bacteremia risk factor^a^ | 46 (13.9) | 431 (8.7) | 1.69 (1.22-2.34) | .002 |  |
| Blood culture obtained in ED | 82 (24.7) | 495 (10.0) | 2.95 (2.26-3.85) | <.001 |  |
| Blood culture obtained in ED from a central venous catheter, peripherally inserted central catheter, or a subcutaneous port (vs peripheral) | 3 (3.7) | 19 (4.0) | 0.92 (0.27-3.18) | .89 |  |
| Blood culture growing any organism^b^ | 23 (26.7) | 88 (17.7) | 1.69 (1.00-2.88) | .05 |  |
| **Blood culture results**^b^ |  |  |  |  |  |
| Coagulase-negative *Staphylococcus* | 17 (19.8) | 44 (8.9) | 2.53 (1.37-4.68) | .003 |  |
| *Staphylococcus aureus* | 3 (3.5) | 8 (1.6) | 2.20 (0.57-8.48) | .25 |  |
| *Escherichia coli* | 1 (1.2) | 12 (2.4) | 0.47 (0.06-3.70) | .48 |  |
| *Klebsiella pneumoniae* | 1 (1.2) | 4 (0.8) | 1.45 (0.16-13.10) | .74 |  |
| Propionibacterium | 1 (1.2) | 0 (0) | NA | .99 |  |
| *Corynebacterium* spp | 0 (0) | 6 (1.2) | NA | .99 |  |
| *Streptococcus viridans* | 0 (0) | 5 (1.0) | NA | .99 |  |
| Other organisms | 1 (1.2) | 10 (2.0) | 0.57 (0.07-4.52) | .60 |  |
| **Antibiotics administered in the ED** |  |  |  |  |  |
| Azithromycin | 20 (6.0) | 140 (2.8) | 2.20 (1.36-3.57) | .001 |  |
| Ceftriaxone | 36 (10.8) | 194 (3.9) | 2.98 (2.05-4.33) | <.001 |  |
| Cephalexin | 2 (0.6) | 29 (0.6) | 1.03 (0.24-4.33) | .97 |  |
| Ciprofloxacin | 7 (2.1) | 126 (2.6) | 0.82 (0.38-1.78) | .62 |  |
| Clindamycin | 3 (0.9) | 21 (0.4) | 2.14 (0.63-7.21) | .21 |  |
| Doxycycline | 1 (0.3) | 15 (0.3) | 0.99 (0.13-7.54) | >.99 |  |
| Levofloxacin | 2 (0.6) | 25 (0.5) | 1.19 (0.28-5.06) | .81 |  |
| Metronidazole | 0 (0) | 49 (1.0) | NA | NA |  |
| Piperacillin-tazobactam | 24 (2.3) | 125 (2.5) | 3.01 (1.91-4.72) | <.001 |  |
| Trimethoprim-sulfamethoxazole | 2 (0.6) | 26 (0.5) | 1.15 (0.27-4.85) | .85 |  |
| Vancomycin | 23 (6.9) | 122 (2.5) | 2.94 (1.86-4.66) | <.001 |  |
| Any antibiotic (listed above) | 79 (23.8) | 569 (11.5) | 2.40 (1.84-3.14) | <.001 |  |
| **Triage data** |  |  |  |  |  |
| Tobacco use in past year | 92 (41.3) (n=223) | 305 (20.5) (n=1,489) | 2.73 (2.03-3.66) | <.001 |  |
| Abuse screen unsafe at home | 14 (5.3) (n=266) | 34 (1.2) (n=2,935) | 4.74 (2.51-8.95) | <.001 |  |
| Abuse consult needed | 29 (11.6) (n=250) | 47 (2.9) (n=1,638) | 4.44 (2.74-7.21) | <.001 |  |
| Homicidal ideation | 6 (2.0) (n=297) | 22 (0.5) (n=4,623) | 4.31 (1.73-10.72) | .006 |  |
| Primary care physician, No. (%) | 96 (28.9) | 1,961 (39.6) | 0.62 (0.49-0.79) | <.001 |  |
|  |  |  |  |  |  |
| Emergency severity index (scale 1-5), mean (SD) | 2.86 (0.81)  (n=323) | 2.90 (0.74)  (n=4,751) | .92 (.79-1.08) | .31 |  |
| Pain score, 0-10 | 4.73 (3.67) (n=176) | 3.84 (3.59) (n=1,343) | 1.07 (1.03-1.12) | .002 |  |
| Body mass index, mean (SD) | 29.81 (11.68)  (n=308) | 29.36 (8.21)  (n=4,767) | 1.01 (.99-1.02) | .36 |  |
|  |  |  |  |  |  |
| Temperature, mean (SD), °F | 97.73 (1.31)  (n=332) | 97.80 (1.15) (n=4,890) | .95 (.86-1.04) | .25 |  |
| Heart rate, mean (SD), beats/min | 89.73 (16.41)  (n=331) | 84.42 (17.63)  (n=4,900) | 1.02 (1.01-1.02) | <.001 |  |
| Respiratory rate, mean (SD), breaths/min | 18.79 (4.88)  (n=331) | 18.15 (2.74)  (n=4,888) | 1.05 (1.02-1.08) | <.001 |  |
| Mean arterial pressure, mean (SD), mmHg | 101.75 (22.01)  (n=330) | 102.41 (17.60) (n=4,891) | 1.00 (.99-1.00) | .52 |  |
| Oxygen saturation, mean (SD), % | 97.55 (2.51)  (n=331) | 97.55 (2.63)  (n=4,884) | 1.00 (.96-1.04) | >.99 |  |
| Admitted from ED (vs Discharged, Transferred, Against Medical Advice, or Left Without Being Seen  Admitted/observation) No. (%) | 264 (79.5) | 1,835 (37.1) | 6.59 (5.02-8.66) | <.001 |  |
| Admitted to the intensive care or step-down unit, No. (%) | 12 (3.6) | 114 (2.3) | 1.59 (0.87-2.92) | .13 |  |

Abbreviations: %, percent; CI, confidence interval; CFU, colony forming unit; ED, emergency department; °F, degrees Fahrenheit; mmHg, millimeters per mercury; NA, not applicable; No., number; OR, odds ratio; SD, standard deviation.

^a^ Defined as having an ED or inpatient diagnosis that included the terms *catheter, device, prosthetic, shunt, implant, intravascular, graft, PICC, central venous, line, osteomyelitis, endocarditis, septic arthritis,* or *septic joint*.

^b^ Some blood cultures grew >1 organism
