# Supplementary material for: Investigating the association of bed bugs with infectious diseases: A retrospective case-control study
Source: Heliyon. 2021 Oct 1;7(10):e08107. doi: 10.1016/j.heliyon.2021.e08107 (PMC8569396; doi:10.1016/j.heliyon.2021.e08107)
Supplement: Supplement 3 [file mmc3.docx]

**Supplementary Table 3. Antibiotic sensitivity in bacteria from blood cultures**

| **Antibiotic Sensitivity** |  | | |
| --- | --- | --- | --- |
|  | **Bed Bugs** | **No Bed Bugs** | ***P* Value** |
| Amoxicillin-clavulanate | 37.5% (3/8) | 59.3% (16/27) | .29 |
| Ampicillin | 23.1% (3/13) | 40.4% (23/57) | .25 |
| Cefazolin | 25% (2/8) | 62.1% (18/29) | .08 |
| Ciprofloxacin | 71.4% (10/14) | 68.6% (35/51) | .84 |
| Clindamycin | 72.7% (8/11) | 65.6% (21/32) | .67 |
| Erythromycin | 40.0% (4/10) | 45.2% (14/31) | .78 |
| Gentamycin | 92.9% (13/14) | 94.3% (50/53) | .84 |
| Levofloxacin | 83.3% (5/6) | 73.9% (17/23) | .63 |
| Oxacillin | 63.2% (12/19) | 63.6% (35/55) | .97 |
| Penicillin | 18.2% (2/11) | 32.6% (14/43) | .36 |
| Tetracycline | 90.9% (10/11) | 86.7% (26/30) | .71 |
| Vancomycin | 100% (11/11) | 97.5% (39/40) | .99 |
